# Expanding the Reach of Pediatric Transcatheter Pacing

**DOI:** 10.19102/icrm.2021.120408

**Published:** 2021-04-15

**Keywords:** Jugular cutdown, leadless pacing, Micra, transcatheter pacing

## Dr. Chang comments

With advances in pacing technology, the application of newer devices to treat pediatric patients and patients with congenital heart disease (CHD) has naturally followed. Pacing indications as well as patient-specific requirements for and limitations to receiving available or new implantable hardware continue to evolve, with either the adaptation of new devices or the development of novel hardware occurring to meet these unique needs.

Conventional pacemaker implantation in pediatric and CHD patients uses either the transvenous or epicardial lead implant route with subcutaneous or submuscular pockets in either the upper chest or abdominal wall. With decades of experience and generally favorable outcomes data reported in concert with the implantation of these devices, they remain the mainstay of therapy to meet the pacing needs of this patient population. Transvenous pacing lead insertion is generally considered in patients weighing at least 15 to 20 kg. However, in the case of implantable cardioverter-defibrillators (ICDs), many electrophysiologists delay transvenous defibrillator leads until the patient weighs at least 25 to 30 kg. While transvenous lead implantation has been successfully performed in very small patients, including some weighing less than 5 kg,^1,2^ the development of venous occlusion is not inconsequential and the risk for complications and increased morbidity associated with lead revision, replacement, or extraction should not be overlooked either. Much has been done to minimize the surgical invasiveness during epicardial system implantation, which has shortened patient recovery times and reduced rates of perioperative complications; however, less-invasive methods of surgical implantation often restrict the amount of exposed myocardium, which can limit available sites for lead fixation with satisfactory lead function.

Pediatric-specific pacing hardware is under development with the intention of creating, from the ground up, pacing technology and hardware specifically designed to meet the unique anatomic constraints and life-long plans for implantable devices in pediatric patients.^3^ Presently, two devices and implant platforms have been developed, both with the capacity for device delivery into the pericardial space, that show promising early results in animal models.^4–6^ However, human trials with short-, mid-, and long-term follow-up data are lacking and device availability and high technical implant skill level are far from being widespread.

It is within this space and time that leadless pacing has gained substantial interest and stands to grow in its application in pediatric and CHD patients. Experience with leadless pacing in pediatric and CHD patients remains quite novel but still growing among implanters. In fact, most of the implant experience with Micra™ leadless pacemakers (Medtronic, Minneapolis, MN, USA) in pediatric patients has only come to publication since 2019. Hacket et al.^7^ are to be commended for their report detailing their experience with a multidisciplinary approach to Micra™ implantation in an otherwise healthy child with asystole-associated syncope. In this particular case, leadless pacing was considered a first-line treatment option in an individual without contraindications to traditional transvenous or epicardial implant approaches. Previous publications^8–13^ have cited limitations to conventional implants due to patient-related circumstances or medical complexities, thereby driving the decision to pursue leadless pacing as a secondary option. Realistically, leadless pacing will likely continue to gain traction in pediatric and CHD patients and will steadily become a legitimate first-line therapeutic option for which pediatric implanters should collectively pursue experience and proficiency.

Femoral and internal jugular venous approaches have been used during pediatric Micra™ implantation with excellent implant success rates and good postimplant device stability and function. Though our collective experience is still fairly limited, published cases^8–13^ have demonstrated successful and functional leadless pacing from the right ventricle (RV) in pediatric patients across a broad spectrum of sizes. Perhaps the foremost question in pediatric implants is whether the leadless pacemaker is too large to go through the lower vasculature. Hacket et al. provided a nice description of their systematic approach to vessel size assessment with preprocedural ultrasound imaging. Our center recently published our experience with Micra™ implantation in the youngest and smallest pediatric patient (16 kg) to date, based on a review of published literature, and the procedure was accomplished with a conventional femoral venous approach.^8^ This obviously challenges the perceived lower limits of patient and vessel sizes and highlights the elasticity and distensibility of the femoral vasculature. Hacket et al. and other implanters have approached jugular venous access in a hybrid fashion with open surgical cutdown to access, control, and close the vessel before, during, and after Micra™ delivery. Considering this collective experience, it would be reasonable to investigate the feasibility and safety of a surgical femoral venous cutdown approach that would provide similar direct control of the femoral vessel during initial access and dilation; a more conventional implant delivery from the lower vasculature for which the delivery system was designed to facilitate; and primary vessel closure after implantation, thereby reducing risks of bleeding complications.

In the end, we must continue to work toward a goal where the ongoing evolution and advancement of pacing technology parallels and considers the unique constraints in, long-term care requirements associated with, and evolving indications for pacing in young patients and those with repaired or palliated CHD.

Philip M. Chang, md, facc, fhrs (philip.chang@ufl.edu)^1^

^1^University of Florida Health Congenital Heart Center, Gainesville, FL, USA

Dr. Chang reports no conflicts of interest for the published content.

References1.Robledo-NolascoROrtiz-AvalosMRodriguez-DiezGTransvenous pacing in children weighing less than 10 kilogramsPacing Clin Electrophysiol200932 Suppl 1S177181[CrossRef][PubMed]1925008810.1111/j.1540-8159.2008.02276.x2.KontaLChubbMHBostockJRogersJRosenthalETwenty-seven years experience with transvenous pacemaker implantation in children weighing <10 kgCirc Arrhythm Electrophysiol201692e003422[CrossRef][PubMed]2685790810.1161/CIRCEP.115.0034223.DubinAMCannonBCSaarelEVPediatric and Congenital Electrophysiology Society initiative on device needs in pediatric electrophysiologyHeart Rhythm2019164e39e46[CrossRef][PubMed]3057997710.1016/j.hrthm.2018.12.0214.ClarkBCOpfermannJDDavisTDKriegerABerulCISingle-incision percutaneous pericardial ICD lead placement in a piglet modelJ Cardiovasc Electrophysiol201728910981104[CrossRef][PubMed]2856942410.1111/jce.132635.Bar-CohenYSilkaMJHillACMinimally invasive implantation of a micropacemaker into the pericardial spaceCirc Arrhy Electrophysiol2018117e006307[CrossRef][PubMed]10.1161/CIRCEP.118.006307PMC6050018299459296.ClarkBCKumthekarRMassPOpfermannJDBerulCIChronic performance of subxiphoid minimally invasive pericardial Model 20066 pacemaker lead insertion in an infant animal modelJ Interv Card Electrophysiol20205911319[CrossRef][PubMed]3161230110.1007/s10840-019-00626-8PMC72737387.HackettGAzizFSamiiSImundoJRDelivery of a leadless transcatheter pacing system as first-line therapy in a 28-kg pediatric patient through proximal right internal jugular surgical cutdownJ Innov Cardiac Rhythm Manage20211244482448610.19102/icrm.2021.120201PMC8081458339368648.MahendranAKBusseySChangPMLeadless pacemaker implantation in a four-year-old, 16-kg childJ Innov Card Rhythm Manag2020111042574261[CrossRef][PubMed]3312341410.19102/icrm.2020.111002PMC75882359.BreatnachCRDunneLAl-AlawiKOslizlokPKennyDWalshKPLeadless Micra pacemaker use in the pediatric population: device implantation and short-term outcomesPediatr Cardiol2020414683686[CrossRef][PubMed]3185820010.1007/s00246-019-02277-y10.Tejman-YardenSNofEBeinartRLeadless pacemaker implantation in a pediatric patient with prolonged sinus pausesPediatr Cardiol2018394844847[CrossRef][PubMed]2952046610.1007/s00246-018-1832-911.McCantaACMorchiGSTuozoFBerdjisFStarrJPBatraASImplantation of a leadless pacemaker in a pediatric patient with congenital heart diseaseHeart Rhythm Case Rep2018411506509[CrossRef][PubMed]10.1016/j.hrcr.2018.07.012PMC62410383047994712.GallottiRGBiniwaleRShannonKRussellMMooreJPLeadless pacemaker placement in an 18-kilogram child: procedural approach and technical considerationsHeart Rhythm Case Rep2019511555558[CrossRef][PubMed]10.1016/j.hrcr.2019.08.011PMC69262143189057313.CortezDInnovative implantation of a leadless pacemaker in a 19 kg paediatric patient via the right internal jugular veinEuropace201921101542[CrossRef][PubMed]3111485910.1093/europace/euz128

## Drs. Beach and Vinocur discuss

First used in humans in 2013, leadless transcatheter pacing systems (TPSs) have dramatically changed the landscape for the adult pacemaker population. The Micra VR™ (Medtronic) and Nanostim™ (Abbott, Chicago, IL, USA) devices initially led the way, while the Micra AV™ (Medtronic) has further advanced the field by allowing atrioventricular synchrony.

Despite the notable benefits of TPSs, their adoption remains limited in the pediatric population. Reasons for this include the large delivery system [27-French (Fr) outer diameter for the Micra VR™], the potential difficulty in device targeting in a small heart, and the uncertain feasibility of late removal with resulting potential for the device to remain implanted for the better part of a century.^1^ In addition, the only device with atrioventricular synchronous capability (Micra AV™) has limited effectiveness at higher heart rates, which are often physiologic in pediatric patients.^2,3^

Despite these drawbacks, leadless TPSs have been used in carefully selected pediatric patients, often motivated by limited venous access, risk factors for endovascular infection, and/or anticipated time-limited pacing need.^4–9^ In this issue of *The Journal of Innovations in Cardiac Rhythm Management*, Hackett et al. report the implantation of a Micra™ VR TPS in a 28 kg, nine-year-old patient by surgical cutdown of the right internal jugular vein.

The authors provide a detailed technical description of their approach to implantation of the Micra™ VR device in a small child. The specific steps taken to optimize room setup for planned neck access are informative, although, given the bulky delivery system, it may be worthwhile to consider inverted patient positioning (head/foot reversal) as has been described for interventional catheterization procedures.^10^ The authors’ observations of the implications of the limited “unsheathing distance” in small patients are likely to be helpful to implanters more experienced with larger patients.

Due to the femoral vein size revealed by preprocedure ultrasound, the authors selected a right internal jugular vein surgical cutdown approach, whose use has been described rarely in placing these devices.^7^ The desire to minimize delivery system–related complications is laudable. However, femoral veins are quite distensible; a 20% to 50% increase from baseline diameter can be demonstrated with simple bedside maneuvers.^11,12^ Therefore, femoral veins may tolerate large sheaths, even in small children; transcatheter pulmonary valves, for example, have been implanted via percutaneous femoral access with a 22-Fr outer diameter delivery system in children with an average weight of 18 kg.^13^ At any access site, surgical cutdown may reduce the risk of significant bleeding following the procedure; the relative merits of percutaneous versus surgical approaches to the implantation of cardiac devices in pediatric patients are unknown, but the strategy proved quite helpful in this case with a short venotomy-to-heart distance, as adequate unsheathing of the delivery system could only be achieved by withdrawing the sheath entirely from the body.

**Figure 1:**
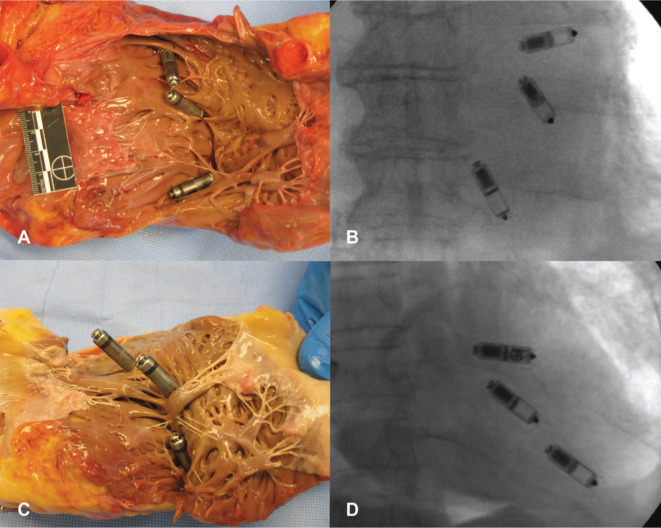
Gross anatomic and fluoroscopic appearance of a multiple simultaneous TPS implantation in a cadaver model using large **(A, B)** and small **(C, D)** adult hearts. Reprinted from Omdahl P, Eggen MD, Bonner MD, Iaizzo PA, Wika K. Right ventricular anatomy can accommodate multiple Micra transcatheter pacemakers. *Pacing Clin Electrophysiol*. 2016;39(4):393–397.^21^

Unlike some reports of leadless TPS implantation in children, this patient did not have contraindications to the implantation of a conventional transvenous device or comorbidities that would make its implantation too risky. The authors cite the ability to allow the patient to continue sports participation, reduced chance for certain complications, minimal cosmetic concerns, and the ability for the device to be shut off in the future as reasons for choosing the system they did. However, alternative implant techniques are available to improve the cosmetic appearance of transvenous devices,^14^ and the need for restriction from competitive sports in addition to during the immediate postprocedure period of lead maturation is still controversial.^15,16^ In 2017, the ICD Sports Safety Registry reported that, in a population of 440 athletes with ICDs, estimated five- and 10-year freedom from definite lead malfunction rates were 95% and 89%, respectively, with no generator malfunctions.^17^ Twenty percent of pediatric athletes experienced lead malfunction over a 10-year period,^18^ which is not notably different from findings in a broader pediatric and young adult cohort.^19^ Thus, apart from ultra–high-contact sports such as boxing, it is likely that this patient could have continued athletics with a transvenous pacemaker, perhaps placed in a subpectoral or axillary position to reduce the risk of system trauma. That being said, the leadless TPS is appealing for this patient’s pacing indication, with abrupt pauses and no need for atrioventricular synchrony at higher heart rates. However, even with a minimal pacing percentage (estimated longevity of 14.5 years per the manufacturer), a Micra VR™ TPS will not come close to satisfying a potentially life-long pacing need in a nine-year-old patient. Serial TPS implantations may be technically feasible **(Figure 1)**, but the clinical merits of this strategy are untested.

In summary, Hackett et al.^20^ eloquently describe their technique for jugular cutdown pediatric leadless TPS implantation in a small child. Use of these systems in carefully selected pediatric patients will continue to increase over time; reports of successful procedures as well as troubleshooting techniques (and perhaps eventually a multicenter registry?) will undoubtedly help to safely advance the field.

Cheyenne M. Beach, md (cheyenne.beach@yale.edu),^1^ and Jeffrey M. Vinocur, md^2^

^1^Department of Pediatrics, Yale University School of Medicine, New Haven, CT, USA

^2^Department of Pediatrics, University of Rochester School of Medicine and Dentistry, Rochester, NY, USA

Drs. Beach and Vinocur report no conflicts of interest for the published content.

References1.GrubmanERitterPEllisCRTo retrieve, or not to retrieve: System revisions with the Micra transcatheter pacemakerHeart Rhythm2017141218011806[CrossRef][PubMed]2871302410.1016/j.hrthm.2017.07.0152.SteinwenderCKhelaeSKGarwegCAtrioventricular synchronous pacing using a leadless ventricular pacemaker: results from the MARVEL 2 StudyJACC Clin Electrophysiol20206194106[CrossRef][PubMed]3170998210.1016/j.jacep.2019.10.0173.von AlvenslebenJCCollinsKKLeadless pacemakers in pediatric patients: is less actually more?J Innov Cardiac Rhythm Manage2020111042634264[CrossRef][PubMed]10.19102/icrm.2020.111003PMC7588237331250054.BreatnachCRDunneLAl-AlawiKOslizlokPKennyDWalshKPLeadless Micra pacemaker use in the pediatric population: device implantation and short-term outcomesPediatr Cardiol2020414683686[CrossRef][PubMed]3185820010.1007/s00246-019-02277-y5.McCantaACMorchiGSTuozoFBerdjisFStarrJPBatraASImplantation of a leadless pacemaker in a pediatric patient with congenital heart diseaseHeartRhythm Case Rep2018411506509[CrossRef][PubMed]3047994710.1016/j.hrcr.2018.07.012PMC62410386.Tejman-YardenSNofEBeinartRLeadless pacemaker implantation in a pediatric patient with prolonged sinus pausesPediatr Cardiol2018394844847[CrossRef][PubMed]2952046610.1007/s00246-018-1832-97.GallottiRGBiniwaleRShannonKRussellMMooreJPLeadless pacemaker placement in an 18-kilogram child: Procedural approach and technical considerationsHeartRhythm Case Rep2019511555558[CrossRef][PubMed]3189057310.1016/j.hrcr.2019.08.011PMC69262148.CortezDInnovative implantation of a leadless pacemaker in a 19 kg paediatric patient via the right internal jugular veinEuropace2019211015421542[CrossRef][PubMed]3111485910.1093/europace/euz1289.MahendranAKBusseySChangPMLeadless pacemaker implantation in a four-year-old, 16-kg childInnov Cardiac Rhythm Manage2020111042574261[CrossRef][PubMed]10.19102/icrm.2020.111002PMC75882353312341410.KubickiRHummelJHöhnRMüllerKStillerBGrohmannJCatheter strategy to ease the procedure and reduce radiation exposure when requiring neck accessOpen Heart202071e001267[CrossRef][PubMed]3259514010.1136/openhrt-2020-001267PMC732251211.SukEHKimDHKilHKKweonTDEffects of reverse Trendelenburg position and inguinal compression on femoral vein cross-sectional area in infants and young childrenAnaesthesia2009644399402[CrossRef][PubMed]1931770510.1111/j.1365-2044.2008.05815.x12.RippeyJCPascuOJacobsIAbdominal compression effectively increases the size of the common femoral vein, as measured by ultrasonographyAnn Emerg Med2008524446452[CrossRef][PubMed]1863218710.1016/j.annemergmed.2008.04.02213.MartinMHShahanavazSPengLFPercutaneous transcatheter pulmonary valve replacement in children weighing less than 20 kgCatheter Cardiovasc Interv2018913485494[CrossRef][PubMed]2919367110.1002/ccd.2743214.RauschCMHughesBHRuncimanMAxillary versus infraclavicular placement for endocardial heart rhythm devices in patients with pediatric and congenital heart diseaseAm J Cardiol20101061116461651[CrossRef][PubMed]2109436810.1016/j.amjcard.2010.07.02515.RostonTMDe SouzaAMSandorGGSSanataniSPottsJEPhysical activity recommendations for patients with electrophysiologic and structural congenital heart disease: a survey of Canadian health care providersPediatr Cardiol201334613741381[CrossRef][PubMed]2343571610.1007/s00246-013-0654-z16.DoRPattonKKCardiovascular implantable electronic devices in athletesCardiovasc Ther2010285327336[CrossRef][PubMed]2055328710.1111/j.1755-5922.2010.00162.x17.LampertROlshanskyBHeidbuchelHSafety of sports for athletes with implantable cardioverter-defibrillators: long-term results of a prospective multinational registryCirculation20171352323102312[PubMed]2858403210.1161/CIRCULATIONAHA.117.02782818.SaarelEVLawIBerulCISafety of sports for young patients with implantable cardioverter-defibrillators: long-term results of the Multinational ICD Sports RegistryCirc Arrhythm Electrophysiol20181111e006305[CrossRef][PubMed]3052034910.1161/CIRCEP.118.00630519.AtallahJEricksonCCCecchinFMulti-institutional study of implantable defibrillator lead performance in children and young adults: results of the Pediatric Lead Extractability and Survival Evaluation (PLEASE) studyCirculation20131272423932402[CrossRef][PubMed]2369496610.1161/CIRCULATIONAHA.112.00112020.HackettGAzizFSamiiSImundoJRDelivery of a leadless transcatheter pacing system as first-line therapy in a 28-kg pediatric patient through proximal right internal jugular surgical cutdownJ Innov Cardiac Rhythm Manage20211244482448610.19102/icrm.2021.120201PMC80814583393686421.OmdahlPEggenMDBonnerMDIaizzoPAWikaKRight ventricular anatomy can accommodate multiple Micra transcatheter pacemakersPacing Clin Electrophysiol2016394393397[CrossRef][PubMed]2671091810.1111/pace.12804PMC4834726

## Dr. Das considers

In the present issue, Hackett et al.^1^ present a case of successful placement of a Micra™ TPS via right internal jugular vein surgical cutdown in a 28-kg pediatric patient. The placement of Micra™ devices in the pediatric population is currently limited using a conventional femoral venous access and even rarer using other venous access approaches due to the accompanying 27-Fr delivery sheath and other issues associated with the small size of children.

I agree with these authors that, with increasing experience and expansion of indications and technology (for example, greater availability of the Micra™ AV device to provide atrioventricular synchrony), the use of this technology in children will become more acceptable as a primary approach. In fact, its progression and acceptance may be akin to the development of percutaneous transcatheter pulmonary valve implantation, which can now be performed in very small children (< 20 kg)^2^ using the same 22-Fr Ensemble™ transcatheter delivery system (Medtronic) used in adults.

The authors performed cardiac magnetic resonance imaging of the RV to reveal an RV end-diastolic volume for comparison with the 0.8-mL volume Micra™ TPS. Pediatric implanters should be aware that, instead of the RV volume, a major limitation may arise due to the 26-mm length of the Micra™ device that, in small RVs, may cause tricuspid valve dysfunction due to impingement of the valve apparatus after septal implant. An additional procedure like magnetic resonance imaging solely to determine RV volume for implant is unnecessary and RV dimensions, including the length from valve annulus to apex, should be obtained by either echocardiography or RV ventriculography in the right anterior oblique view at the time of implant.

**Figure 2:**
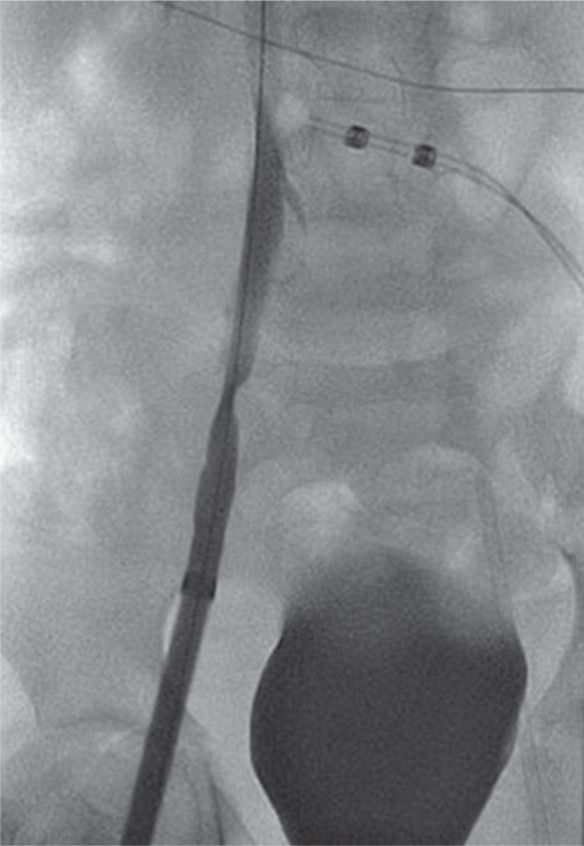
Right iliac venogram with Micra™ delivery sheath showing venous injury without extravasation.

**Figure 3:**
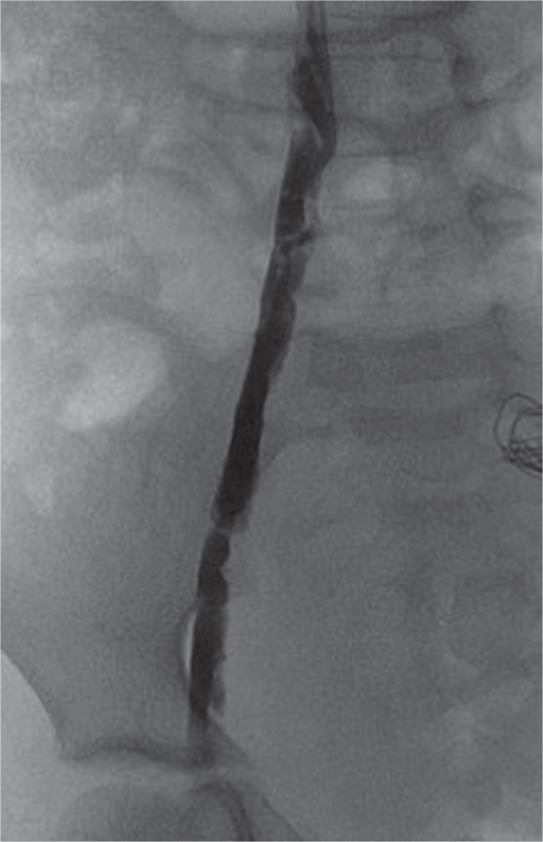
Follow-up angiogram taken the next day showing irregular intima after degloving injury.

While noninvasive venous duplex scans are a useful guide, venous structures are expandable and dimensions may depend upon the intravascular volume status and supine positioning. A similar cutdown approach may be used for femoral venous access as well as for anticipated borderline sizes of veins relative to the delivery system. The 27-Fr vascular access sheath, albeit exceptionally smooth with a lubricious hydrophilic coating, dries up quickly during maneuvering through the access site as a relatively long part remains outside in small children. I had a 20-kg child in whom the dilator of the delivery system could be passed over the wire repeatedly to the right atrium, but passing the delivery system to the inferior vena cava was challenging and caused degloving intimal injury to the iliac vein **(Figures 2 and 3)**.

I congratulate Hackett et al. on the exceptional technical achievement in their case. A femoral venous approach adopts the advantage of the system’s design for successful inferior vena cava approach to the RV. Additionally, an injury to the superior vena cava from upper body venous access may preclude or complicate implantation of a transvenous pacemaker system if Micra™ implantation is unsuccessful.

Srikant Das, md (sdas@uams.edu)^1,2^

^1^Pediatric Cardiology, Arkansas Children’s Hospital, Little Rock, AK, USA

^2^Pediatric Cardiology, University of Arkansas for Medical Sciences, Little Rock, AK, USA

Dr. Das reports no conflicts of interest for the published content.

References1.HackettGAzizFSamiiSImundoJRDelivery of a leadless transcatheter pacing system as first-line therapy in a 28-kg pediatric patient through proximal right internal jugular surgical cutdownJ Innov Cardiac Rhythm Manage20211244482448610.19102/icrm.2021.120201PMC8081458339368642.MartinMHShahanavazSPengLPercutaneous transcatheter pulmonary valve replacement in children weighing less than 20 kgCatheter Cardiovasc Interv2018913485494[CrossRef][PubMed]2919367110.1002/ccd.27432
